# Perioperative serum albumin as a predictor of adverse outcomes in abdominal surgery: prospective cohort hospital based study in Northern Tanzania

**DOI:** 10.1186/s12893-020-00820-w

**Published:** 2020-07-14

**Authors:** Christian Ephata Issangya, David Msuya, Kondo Chilonga, Ayesiga Herman, Elichilia Shao, Febronia Shirima, Elifaraja Naman, Henry Mkumbi, Jeremia Pyuza, Emmanuel Mtui, Leah Anku Sanga, Seif Abdul, Beatrice John Leyaro, Samuel Chugulu

**Affiliations:** 1 Kilimanjaro Christian Medical University College, P.O Box 2240, Moshi, Tanzania; 2 Department of General Surgery, Kilimanjaro Christian Medical Centre, P.O Box 3010, Moshi, Tanzania; 3 Department of Medical Laboratory, Kilimanjaro Christian Medical Centre, P.O Box 3010, Moshi, Tanzania

**Keywords:** Serum albumin, Delta albumin, Adverse outcome, Abdominal surgery

## Abstract

**Background:**

Albumin is an important protein that transports hormones, fatty acids, and exogenous drugs; it also maintains plasma oncotic pressure. Albumin is considered a negative active phase protein because it decreases during injuries and sepsis. In spite of other factors predicting surgical outcomes, the effect of pre and postoperative serum albumin to surgical complications can be assessed by calculating the percentage decrease in albumin (delta albumin). This study aimed to explore perioperative serum albumin as a predictor of adverse outcomes in major abdominal surgeries**.**

**Methods:**

All eligible adult participants from Kilimanjaro Christian Medical Centre Surgical Department were enrolled in a convenient manner. Data were collected using a study questionnaire. Full Blood Count (FBP), serum albumin levels preoperatively and on postoperative day 1 were measured in accordance with Laboratory Standard Operating Procedures (SOP). Data was entered and analyzed using STATA version 14. Association and extent of decrease in albumin levels as a predictor of surgical site infection (SSI), delayed wound healing and death within 30 days of surgery was determined using ordinal logistic regression models. In determining the diagnostic accuracy, a Non-parametric Receiver Operating Curve (ROC) model was used. We adjusted for ASA classification, which had a negative confounding effect on the predictive power of the percent drop in albumin to adverse outcomes.

**Results:**

Sixty one participants were studied; the mean age was 51.6 (SD16.3), the majorities were males 40 (65.6%) and post-operative adverse outcomes were experienced by 28 (45.9%) participants. In preoperative serum albumin values, 40 (67.8%) had lower than 3.4 g/l while 51 (91%) had postoperative albumin values lower than 3.4 g/l. Only 15 (27.3%) had high delta albumin with the median percentage value of 14.77%. Delta albumin was an independent significant factor associated with adverse outcome (OR: 6.68; 95% CI: 1.59, 28.09); with a good predictive power and area under ROC curve (AUC) of 0.72 (95% CI 0.55 0.89). The best cutoff value was 11.61% with a sensitivity of 76.92% and specificity of 51.72%.

**Conclusion:**

Early perioperative decreases in serum albumin levels may be a good, simple and cost effective tool to predict adverse outcomes in major abdominal surgeries.

## Background

Abdominal surgery is a common surgical procedure. It is performed as either elective surgery or on an emergency basis. Underlying diseases may cause stress to body tissues and surgical trauma adds additional stress that triggers a corrective response by the body [1].

Albumin is the major protein of human plasma. It constitutes approximately 60% of the total plasma protein and its normal serum concentration is 3.5–5.0 g/dl. A serum level of less than 3.4 g/dl is considered hypoalbuminemia (citation). Plasma albumin has three primary functions: osmotic, transportation and nutritional and it accounts for more than 75–80% of total plasma osmotic pressure (25 mmHg). During physiological stress, decreases in serum albumin levels to hypoalbuminemia levels causes a fall in oncotic pressure, which in turn leads to interstitial oedema [2].

Studies have shown that hypoalbuminemia contributes negatively to the n process of wound healing, fracture union and severity of disease [2]. Hypoalbuminemia is linked to mortality and postoperative complications such as surgical site infections (SSI) and reoperations together with longer hospital stays [3, 4]. Additionally, one study reported that early decreases in serum albumin levels of ≥10 g/dl in postoperative day one presents a three- fold increased risk in postoperative adverse complications in major abdominal surgeries. In another study, a decrease of albumin of > 15% from the mean value of 3.94 g/dl was associated with longer hospital stays, SSI and higher risk of reoperation. In an oesophagestomy study related to malignancy, patients with postoperative low albumin had higher chances of anastomotic leaks and mortality within 7 days of surgery [5].

Different cutoff points of percentage decrease of perioperative albumin (delta albumin) has been proposed, some with 15% and others with 24.77%; however generally the higher percentage the higher odds of postoperative adverse complications with good predictive value [1, 6, 7].

Serum albumin is simple and easily interpreted biomarker with valuable impact in perioperative period; however in our setting it is not a routine investigation for preoperative assessment. Understanding the correlation and the strength of association of decrease serum albumin and surgical adverse complications will also improve management protocol at the department of surgery and help to reduce burden of healthcare expenditure by improvement of patient care and proper management of surgical resources.

## Methods

### Study design and setting

This was a prospective cohort study conducted from October 2018 to March 2019 at the Department of General Surgery at KCMC in Moshi in the Kilimanjaro region. KCMC is a Northern Zone Consultant Hospital in Tanzania. The hospital receives referred patients from northern and central regions namely Arusha, Manyara, Tanga, Dodoma, Singida and districts from the Kilimanjaro region. The population served is more than 15 million people.

### Study population

The study population consisted of patients aged 18 years and above, admitted for either elective or emergency reasons and indicated for major abdominal surgery. All had consented for the study.

### Sample size and sampling procedure

All eligible patients were included in the final sample. A total of 144 patients were expected to be operated representing an average of 6 patients per week over 6 months. However, based on eligibility criteria, a total of 61 patients were included in the final sample. We conveniently enrolled participants after completing the process of informed consent.

### Definitions of outcome measures

The outcomes of interest were SSI, delayed wound healing and death within 30 days post -surgery. SSI was measured as wound infection within 3 days post-surgery, delayed wound healing was counted as a prolongation of healing for more than 7 days due to the interruption of the normal process of wound healing and death within 30 days post-surgery. When a participant experienced one of these named outcomes, it was measured as an adverse outcome.

### Clinical characteristics

Laboratory parameters were serum albumin levels on the preoperative day and on postoperative day 1 and full blood count (FBC).

### Data collection methods, tools and study procedures

We used a questionnaire to collect a patient’s demographic information, including age, sex and level of education. Laboratory parameters were processed in accordance with Standard Operating Procedures. A blood sample of at least 2mls was collected from participants. Samples were stored at the temperature range 20–25 °C and serum was separated immediately from the clot. Analysis of the specimen was done promptly. COBAS Integra 400 Plus was used for specimen analysis with an interpretation of expected normal adult serum albumin in the range of 3.5–5.0 g/dl. Preoperative serum albumin was collected then followed by postoperative serum albumin on day 1. The percentage change of albumin values (Delta Albumin) was calculated using formula ({POD-POD1}/POD × 100). The reference percentage change observed was approximately 14.77% as adopted from a study done in China above which was associated with adverse outcomes [4]. Intraoperative data from patient case notes was followed up. Participants were followed up during the period 24 h to 30 days post-surgery to evaluate for adverse surgical outcomes such as death, SSI, delayed wound healing and length of hospital stay. This was done at general surgery wards before discharge. After discharge, it was done on days 7, 14, 21 and 30 at a Surgical Outpatient Department (SOPD). An appointment was made by phone. Variables for the study were obtained from patient data and case notes as in the data sheet.

### Data management and analysis plan

Data were entered, cleaned and analyzed by using STATA version 14. Descriptive statistics for the patient’s characteristics were summarized using frequencies and proportions for categorical variables while measure of central tendency and their respective measure of dispersion were used to summarize continuous variables depending on their distribution. Logistic regression was used to check association between different levels of decrease in serum albumin and adverse surgical outcomes. Significance was considered at 95% confidence interval and p-vale < 0.05. In determining the diagnostic accuracy, a Non-parametric Receiver operating curve (ROC) model was used. We adjusted for ASA classification, which had a negative confounding effect on the predictive power of percent drop in albumin to adverse outcomes.

## Results

### Socio-demographic and clinical characteristics

Among all (61) study participants, the mean age was 51.6 ± 16.3 with the majority 34 (55.7%) being aged 50 years and above. There were 40 males (65.6%) and the majority, 42 (68.9%) had attained primary education. Forty (65.6%) participants had an emergency surgical indication and 28 (45.9%) had post-operative adverse outcomes (Table 1).

**Table 1:** Socio-demographic and clinical characteristics of participants (*N* = 61)

Characteristics	N	%
**Age (years**)		
18–24	5	8.2
25–49	22	36.1
50+	34	55.7
*Mean ± SD (51.6 ± 16.3)*		
**Sex**		
Male	40	65.6
Female	21	34.4
**Education level**		
Non formal	4	6.5
Primary level	42	68.9
Secondary and above	15	24.6
**Surgical indication**		
Emergency	40	65.6
Elective	21	34.4
**American Society of Anesthesia classification (*n*** **= 52)**		
1	21	40.4
2	31	59.6
**Length of surgery (minutes) (*n*** **= 61)**		
0–120	15	24.6
121 & above	46	75.4
**Postoperative adverse outcome**		
No	33	54.1
Yes	28	45.9
**Types of surgeries**		
Exploratory laparotomy	42	68.8
Bypass & anastomosis procedures	11	18.0
Colostomy procedures	4	6.6
Oncological procedures	2	3.3
Biliary surgeries	2	3.3

### Laboratory tests

As summarized in Table 2 below, participants were tested for preoperative and postoperative serum albumin levels. The percentage change of their albumin value was calculated using the formula ({POD-POD1}/POD ×100). Among 59 participants with preoperative serum albumin values, 40 (67.8%) had lower than 3.4 g/l while out of 56 participants, 51 (91%) had postoperative albumin values lower than 3.4 g/l. Out of 55 participants, 28 (50.9%) had a high change in percentage difference between pre and postoperative (delta albumin) with the median percentage value of 14.77%. Twenty-four participants had normal hematocrit levels and only one participant had a low white blood cell count.

**Table 2:** Laboratory tests of participants (*N* = 61)

Characteristics	N	%
**Pre- operative albumin level (g/L) (*n*** **= 59)**		
< 3.4	40	67.8
3.4–5.0	19	32.2
*Mean ± SD (2.9 ± 9.8)*		
**Post- operative albumin level (g/L) (*n*** **= 56)**		
< 3.4	51	91.1
3.4–5.0	5	8.9
*Mean ± SD (2.5 ± 7.1)*		
**Delta albumin (%) (*n*** **= 55)**		
< 14.77	27	49.1
14.77 and above	28	50.9
**HCT count (*n*** **= 60)**		
Low	22	36.7
Normal	24	40.0
High	14	23.3
**WBC count (*n*** **= 60)**		
Below normal range	1	1.6
Normal & above	60	98.4

### Distribution of adverse outcomes associated with low and high delta albumin participants

**Fig. 1:**
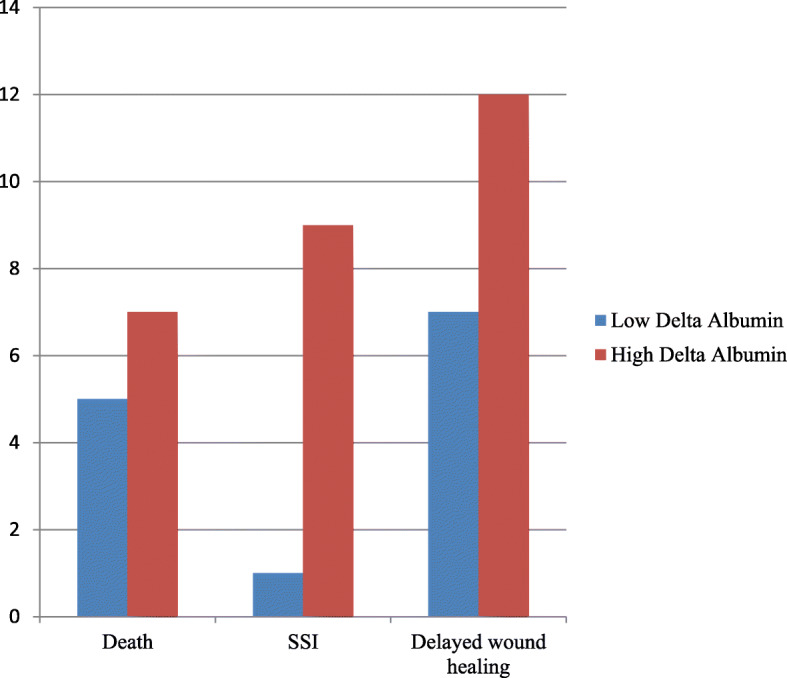
Distribution of adverse surgical outcomes by delta albumin

Participants with a high percentage change in perioperative albumin had a higher likelihood of delayed wound healing, death with 30 days and significant chance of developing surgical site infection, as illustrated in Fig. 1.

### Factors associated with post-operative adverse outcomes

Among 61 study participants, the prevalence of post-operative adverse outcome was 45.9%. Also 61% of those with postoperative adverse outcome had high delta albumin. When adjusted for other factors, participants’ aged 25–49 years had almost 9.6 times higher odds of adverse outcome compared to those aged 18–24 years (OR: 9.62; 95% CI: 0.52, 177.36) while being female was 67% protective against an adverse outcome as compared to their male counterparts (OR: 0.33; 95% CI: 0.05, 1.37). Those participants with more than 2 h of operative time had a 73% higher chance of an adverse surgical outcome compared to those with less operative time. (OR: 1.73; 95% CI: 0.35 8.45); however participants with higher percentage change in pre and post-operative albumin level (delta albumin) had a significant chance of developing adverse outcomes compared to those with low delta albumin (OR: 6.68; 95% CI: 1.59, 28.09) with the *p*-value 0.01(Table 3).

**Table 3:** Factors associated with postoperative adverse outcome

Factors	Post-OP Adverse Outcome %	COR	(95% CI)	***P-***value	AOR	(95% CI)	***P-***value
**Age (years)**							
18–24	20.0	1			1		
25–49	59.1	5.77	(0.55, 60.60)	0.144	9.62	(0.52, 177.36)	0.128
50+	41.2	2.80	(0.28, 27.79)	0.379	5.95	(0.34, 104.06)	0.222
**Sex**							
Male	55.0	1			1		
Female	28.6	0.33	(0.11, 1.01)	0.053	0.26	(0.05, 1.37)	0.112
**Delta albumin (%)**							
< 14.77	33.3	1			1		
14.77 and above	60.7	3.09	(1.03, 9.31)	0.45	6.68	(1.59, 28.09)	0.010
**Surgical indication**							
Emergency	50.0	1			1		
Elective	38.1	0.62	(0.21, 1.81)	0.377	1.28	(0.27, 6.11)	0.759
**ASA**							
1	47.6	1			1		
2	38.7	0.69	(0.23, 2.13)	0.524	0.44	(0.10, 2.12)	0.289
**Length of surgery (min)**							
0–120	38.9	1			1		
120 and above	48.8	1.50	(0.49, 4.60)	0.478	1.73	(0.35 8.45)	0.500

*Note: *COR* Crude odds ratio, *AOR* Adjusted odds ratio

### Predictive accuracy of delta albumin to adverse surgical outcome

**Fig. 2:**
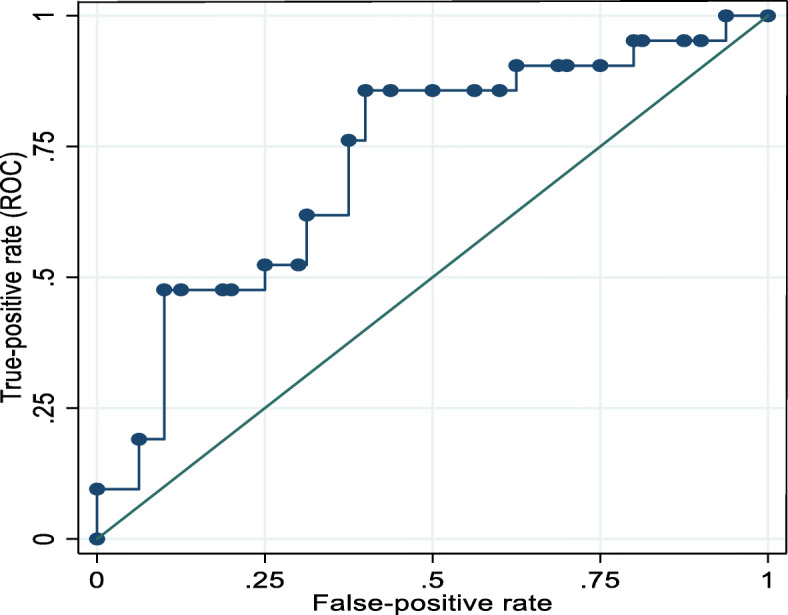
Area under receiver operating characteristics curve for predicting adverse outcomes using delta albumin while adjusting for ASA classification of participants from October 2018 to March 2019

The area under receiver operating characteristic curve (AUC) of 0.72 (95% CI 0.55, 0.89) was obtained, which signifies a good predictive power of adverse surgical outcome for delta albumin. The best cutoff value was 11.61% with the sensitivity of 76.92% and specificity of 51.72% (Fig. 2).

## Discussion

In this study, the majority of participants underwent major abdominal surgery, and had lower preoperative and postoperative serum albumin level than the standard lower limit. The mean postoperative albumin was even lower than the mean preoperative value. This observation supports the theory of a decrease in albumin level due to inflammation and surgical trauma [8]. Similarly, a reduction in albumin levels after surgical trauma is related to generalized inflammation, or systemic inflammatory response syndrome, which is characterized by increased capillary leakage of albumin. Additionally, in the study of early decreases in postoperative albumin as predictor of complications, there was a significant decrease in postoperative albumin levels. However, the final analysis did not suggest its predictive role.

In contrast to this study, in which preoperative serum albumin levels were not an independent factor for adverse outcome was another study in which preoperative low albumin was an independent and significant risk factor for adverse surgical outcome [8]. This may be associated with the involvement of only colorectal cancer patients in the study. These patients were at a higher risk of malnutrition due to cancer-induced metabolism, lower dietary intake and the effect of tumor necrosis factor-alpha on alteration of liver protein production.

The percentage decrease of serum albumin (delta albumin) after surgery was higher in participant with even lower postoperative albumin levels. In this study, delta albumin was associated with adverse postoperative surgical site infection, delayed wound healing and death within 30 days. These findings correlate with a study done in Australia to evaluate the relationship between the value of delta albumin and surgical complications in patients who underwent bowel resection due to Crohn’s disease. Higher delta albumin levels were significantly associated with postoperative complications, namely intra-abdominal abscess, anastomotic leak, reoperation and death. Neither preoperative nor postoperative serum albumin was an independent risk factor for surgical complications [9]. Despite differences in ethnicity between the Australian participants and our participants, similar methods were used in calculating the percentage difference of albumin levels and the correlation with postoperative outcomes.

The delta albumin (ΔAlb) was an independent risk factor for severe complications in CRC patients after curative laparoscopic surgery [3]. A study done in Thailand also revealed similar findings that hypoalbuminemia is a potential predictor of delayed recovery of bowel function postoperatively and significantly associated with postoperative complications [4]. In both cohorts, there were similar risks of hypoalbuminemia due to malignancy, which were depicted in similar outcome patterns.

In this cohort, postoperative adverse outcomes were evident in approximately 46%. These included surgical site infections, delayed wound healing and death with 30 days post operatively. However, other factors were also associated with surgical outcome: operation time of more than 2 h was 73% suggestive of poorer outcome than with shorter surgeries. This may be due to longer surgical trauma, longer inflammatory phase post-surgery and higher risk of delay recovery time.

Additionally, patients who underwent elective operations had a protective effect of 40% compared to emergency cases. Elective patients were clinically more stable with a fair nutritional status. This observation may be attributed to a less morbid condition of elective patients. In rectal surgery, hypoalbuminemia combined with higher ASA classification was significantly associated with wound infection, remote infections such as pneumonia, and anastomotic leakage. Other complications included reoperation and death [10]. This may be attributed to comorbid situation such as hypertensive heart disease and diabetic mellitus, together with operative stress.

In light of how accurately the decrease in albumin level would predict an adverse outcome, delta albumin was associated with surgical site infection. An area under receiver operating characteristic curve (AUC) of 0.72 (95% CI 0.55 0.89) was obtained, which is evidence of good predictive power. The best cutoff value was 11.61% with a sensitivity of 76.92% and specificity of 51.72%.This explains how good perioperative decreases in serum albumin are a predictor for potential surgical complications with 30 days of surgery. The findings were consistent with other studies in which delta albumin of more than 15% was a cutoff point with higher sensitivity and specificity [3]. Also, in studies done in China, Thailand and Australia, the cutoff point of 15.0, 13.2, and 24.27% were associated with larger AUC and high sensitivity and specificity. These similar findings render the predictive accuracy of a decrease of serum albumin as a predictor of adverse surgical outcome [1, 3, 9].

We conducted a prospective study where individual follow-up of each study participant, both preoperatively and postoperatively was done. This maximized the reliability of collected information as there was less recording and recall biases. However the study was conducted in a single institution. This may not represent a true sample representation of the developing world. The recruitment time for participants was as short as 6 months. The number of participants was small. This may affect the power of this study. This study did not take into account the impacts of liver function and body fluid volume on serum albumin concentrations. An adequate function of the hepatocytes is a prerequisite to the production of normal serum albumin levels. Therefore, a preoperative assessment of liver function tests would exclude confounding liver diseases as well as explaining a low preoperative albumin. Therefore abnormal liver function tests would provide important exclusion criteria for the studied patients.

## Conclusion

This study showed that an early perioperative decrease in serum albumin level predicts postoperative adverse outcomes in patients undergoing major abdominal surgeries. The surgical care team is advised to be aware of the percentage decrease of serum albumin in the early postoperative period, even for patients with normal preoperative serum albumin levels. Therefore serum albumin may be used as a simple and low-cost prognostic tool to predict the risk of adverse surgical outcomes. Nevertheless, multiple limitations present the opportunity for more precise studies in order to solidify the revealed association.

## Data Availability

All data and materials concerning this research article are available for sharing if needed.

## Abbreviations

ASA: American Society of Anesthesiologists
AUROCC: Area Under Receiver Characteristic Curve
CRC: Colorectal Cancer
CRP: C-reactive Protein
DM: Diabetes Mellitus
FBC: Full Blood Count
HCT: Hematocrit
KCMC: Kilimanjaro Christian Medical Centre
KCMUCo: Kilimanjaro Christian Medical University College
kDa: Kilo Dalton
POD: Postoperative Day
SSI: Surgical Site Infection
TB: Tuberculosis
WBC: White Blood Count
ΔAlb: Change in Serum Albumin
